# Effects of dietary NDF/NFC ratios on *in vitro* rumen fermentation, methane emission, and microbial community composition

**DOI:** 10.3389/fvets.2025.1588357

**Published:** 2025-06-24

**Authors:** Jichao Li, Feng Guan, Pengyu Liu, Huiting Ma, Jiyou Zhang, Yumin Ma, Shengyong Mao, Xiao’e Xiang, Wei Jin

**Affiliations:** ^1^Laboratory of Gastrointestinal Microbiology, Jiangsu Key Laboratory of Gastrointestinal Nutrition and Animal Health, National Center for International Research on Animal Gut Nutrition, College of Animal Science and Technology, Nanjing Agricultural University, Nanjing, China; ^2^Centre for Ruminant Nutrition and Cleaner Production Innovation, College of Animal Science and Technology, Nanjing Agricultural University, Nanjing, China; ^3^Jiangsu Jiahui Biotechnology Co., Ltd., Haian, China; ^4^National Experimental Teaching Center for Animal Science, Nanjing Agricultural University, Nanjing, China

**Keywords:** NDF/NFC ratios, *in vitro* rumen fermentation, methane emission, bacterial community composition, archaeal community composition

## Abstract

The objective of this study was to investigate the effects of dietary neutral detergent fiber (NDF) to non-fibrous carbohydrate (NFC) ratios on *in vitro* rumen fermentation, methane production, and microbiota in dairy cows. *In vitro* rumen fermentation was conducted with five dietary groups: R0.48 (NDF/NFC = 0.48), R0.57 (NDF/NFC = 0.57), R0.70 (NDF/NFC = 0.70), R0.90 (NDF/NFC = 0.90), and R1.12 (NDF/NFC = 1.12). As the NDF/NFC ratios increased, total gas production decreased linearly. The degradation rates of dry matter (DM), neutral detergent fiber (NDF), and acid detergent fiber (ADF) decreased, showing a quadratic response (*p* = 0.018). Methane production per unit of DM was not significantly affected (*p* > 0.05), whereas methane production per unit of degraded DM increased (*p* < 0.05). The concentrations of acetate, lactate, and the acetate-to-propionate ratio, as well as pH, increased linearly (*p* < 0.05), whereas the concentrations of propionate, isobutyrate, isovalerate, and total volatile fatty acids (TVFA) decreased linearly (*p* < 0.05). Microbial crude protein (MCP) production was greater in the low NDF/NFC groups (*p* = 0.003). Quantitative real-time PCR analysis revealed that anaerobic fungi were more abundant in the high NDF/NFC groups (*p* = 0.001), whereas bacterial and archaeal abundances did not differ significantly among groups (*p* > 0.05). Illumina MiSeq PE250 sequencing revealed that the alpha diversity of both bacterial and archaeal communities was influenced by NDF/NFC (*p* < 0.05). Principal coordinate analysis further indicated that composition of bacterial (*R*^2^ = 0.298, *p* = 0.001) and archaeal (*R*^2^ = 0.470, *p* = 0.001) communities differed significantly among groups. Differences in bacterial communities were primarily driven by Firmicutes (e.g., *Ruminococcus*, *Pseudobutyrivibrio*) and Proteobacteria (e.g., *Succinivibrio*, *Ruminobacter*), whereas variations in archaeal communities were associated with Methanobacteriales and Methanomicrobiales. In conclusion, increasing dietary NDF/NFC ratios led to higher methane production per unit of degraded DM, reduced nutrient degradation, and lower TVFA concentrations during *in vitro* rumen fermentation, accompanied by distinct shifts in bacterial and archaeal community composition.

## 1 Introduction

Methane (CH_4_) emissions from livestock account for approximately 30% of global anthropogenic methane output (1). Among these sources, ruminants are the major contributors, with the rumen microbiota responsible for around 18% of total anthropogenic methane emissions (2). Moreover, methane production represents a loss of 2–12% of the gross energy intake in ruminants (3). Therefore, mitigating methane emissions from ruminants is critical for reducing global greenhouse gas emissions. Diet plays a pivotal role in modulating methane production within the gastrointestinal tract of ruminants. The relationship between dietary composition and methane emissions has been extensively investigated, with particular focus on the influence of carbohydrate sources and the forage-to-concentrate ratio. Several *in vitro* studies have examined the impact of dietary concentrate-to-roughage ratios on methane production. For example, after 24 h of *in vitro* rumen fermentation, a diet containing 70% forage resulted in lower methane production and total volatile fatty acid (TVFA) concentrations compared to a diet with 30% forage (4). In contrast, another study reported that a high-forage diet (70%) reduced total gas production but increased methane output relative to a low-forage diet (30%) following 24 h of fermentation (5). Similarly, methane production increased from 701 μmol to 754 μmol, and then to 812 μmol, as the forage proportion decreased from 80 to 50 and 20%, respectively, accompanied by a linear increase in TVFA concentrations (6). After 72 h of *in vitro* fermentation, a high-fiber diet (comprising 70% corn silage and 30% concentrate) led to a lower molar proportion of methane and higher concentrations of short-chain fatty acids compared to a high-concentrate diet (30% corn silage and 70% concentrate) (7). Consistent findings were observed in two *in vivo* studies on lactating cows, where high-forage diets (53.3 and 65%) resulted in greater methane emissions (430 vs. 399 g/day and 492 vs. 404 g/day, respectively) compared to low-forage diets (37.4 and 35%) (8, 9). However, a study on goats found no significant differences in methane emissions or TVFA concentrations between diets containing 41 and 58% forage (10). In another study involving Charolais cross heifers, methane emissions were highest in animals fed a diet with 40% forage compared to those receiving diets with 65% or 10% forage (11).

The conventional classification of ruminant diets based on forage-to-concentrate ratios lacks precision and offers limited nutritional guidance for effectively mitigating enteric methane emissions. Carbohydrates, the primary constituents of ruminant diets, are fermented in the rumen to supply energy while generating key precursors for methanogenesis, notably hydrogen and carbon dioxide (2). Dietary interventions, such as manipulation of feed composition or inclusion of bioactive compounds could modulate host–microbe interactions, nutrient digestibility, and metabolic pathways, thereby influencing microbial community dynamics and energy utilization in livestock (12–14). Among dietary carbohydrate indices, neutral detergent fiber (NDF) and non-fiber carbohydrates (NFC) provide a more nuanced classification, representing slowly and rapidly fermentable carbohydrate fractions, respectively. This classification is especially useful for designing rations aimed at reducing methane emissions. Despite their nutritional significance, studies investigating the mechanistic link between the NDF/NFC ratio and methane production remain limited. Therefore, this study aimed to evaluate the effects of varying NDF/NFC ratios on *in vitro* rumen fermentation, methane production, and microbial community composition in dairy cows. The results are expected to provide a theoretical foundation for developing precision low-methane diets using the NDF/NFC ratio as a key nutritional strategy.

## 2 Materials and methods

### 2.1 Experimental design

The *in vitro* rumen fermentation experiment was conducted using a completely randomized design with five dietary treatments: R_0.48_ (NDF/NFC = 0.48), R_0.57_ (NDF/NFC = 0.57), R_0.70_ (NDF/NFC = 0.70), R_0.90_ (NDF/NFC = 0.90), and R_1.12_ (NDF/NFC = 1.12). The dietary composition and nutrient levels are detailed in Table 1. For each treatment, six replicates were included, and three independent incubation runs were performed at different time points (15). Each incubation run consisted of 36 samples in total: 30 treatment samples and 6 blanks (containing only the inoculum). The experimental procedures closely followed the methods described by Liu et al. (16).

**Table 1:** Composition and nutrient levels of experimental diets.

Items			Groups^a^		
	R_0.48_	R_0.57_	R_0.70_	R_0.90_	R_1.12_
Ingredients (% of dry matter)					
Corn	46.00	37.00	30.00	20.40	11.50
Soybean meal	20.00	18.50	16.00	14.40	10.00
DDGS	4.00	4.50	4.00	5.20	8.50
Corn silage	15.00	22.50	23.00	25.80	29.50
Alfalfa hay	5.00	8.50	17.00	21.20	28.50
Rice Straw	9.90	8.90	9.90	12.90	11.90
Premix^b^	0.10	0.10	0.10	0.10	0.10
Total	100.00	100.00	100.00	100.00	100.00
Nutrient levels (% of dry matter, unless noted)					
Dry matter	68.07	62.83	62.80	61.20	58.83
Crude protein	16.64	16.62	16.61	16.64	16.61
NE_L_ (Mcal/kg)^c^	1.64	1.60	1.53	1.47	1.43
Ether extract	2.84	2.90	2.81	2.84	2.88
Neutral detergent fiber	22.88	25.85	31.22	36.87	40.69
Acid detergent fiber	11.38	13.15	18.04	21.98	26.09
Ash	4.48	4.91	5.48	6.19	6.67
Calcium	0.25	0.32	0.44	0.51	0.62
Phosphorus	0.37	0.36	0.34	0.32	0.32
NFC^d^	53.16	49.72	43.88	37.46	33.15
NDF/NFC	0.48	0.57	0.70	0.90	1.12

a R_0.48_ (NDF/NFC = 0.48), R_0.57_ (NDF/NFC = 0.57), R_0.70_ (NDF/NFC = 0.70), R_0.90_ (NDF/NFC = 0.90) and R1.12 (NDF/NFC = 1.12).

b Premix, including (per kg of DM) 400,000 IU of vitamin A, 320,000 IU of vitamin D3, 1,200 IU of vitamin E, 1400 mg of Cu, 12,000 mg of Zn, 60,000 mg of Fe, 12,000 mg of Mn, 40 mg of Se, 400 mg of I, 160 mg of Co, 28% of Ca and 5.4% of P.

c NEL = net energy for lactation and calculated according to NRC (17).

d NFC (%) = 100 – (NDF + CP + EE + ash), NFC, non-fibrous carbohydrate.

### 2.2 *In vitro* incubation

Rumen fluid was obtained from three healthy Holstein dairy cows in mid-lactation. The cows were fed a diet formulated according to NRC (2001) requirements (17), including 9.05% corn meal, 2.26% barley, 3.62% soybean meal, 4.52% double low rapeseed meal, 1.58% distiller dried grains with solubles, 6.79% brewers wet grain, 2.71% beet pulp pellet, 56.56% corn silage, 6.79% alfalfa hay, 2.44% oat hay, 2.26% molasses, 0.27% NaHCO_3_, 1.13% premix (Vitamin and mineral mix contained the following ingredients per kilogram of diet: vitamin A, 22.5 KIU/kg; vitamin D3, 5.0 KIU/kg; vitamin E, 37.5 IU/kg; vitamin K3, 5.0 mg/kg; Mn, 63.5 mg/kg; Zn, 111.9 mg/kg; Cu, 25.6 mg/kg; and Fe, 159.3 mg/kg.) and contained dry matter (DM) 47.80%, crude protein (CP) 15.90%, ether extract (EE) 3.53%, neutral detergent fiber (NDF) 31.46%, acid detergent fiber (ADF) 18.70%, crude ash 7.06% on a dry matter basis. Rumen fluid was collected approximately 2 h before the morning feeding, with 500 mL obtained from each donor cow. The fluids were pooled, filtered through four layers of cheesecloth, and immediately mixed with buffer solution at a 1:2 (v/v) ratio under anaerobic conditions in a 39°C water bath. The buffer, identical across all treatments, was prepared according to Menke and Steingass (18) and contained 8.75 g NaHCO_3_, 1.00 g NH_4_HCO_3_, 1.43 g Na_2_HPO_4_, 1.55 g KH_2_PO_4_, 0.15 g MgSO_4_·7H_2_O, 0.52 g Na_2_S, 0.017 g CaCl_2_·2H_2_O, 0.015 g MnCl_2_·4H_2_O, 0.002 g CoCl_2_·6H_2_O, 0.012 g FeCl_3_·6H_2_O, and 1.25 mg resazurin per liter (18). A 100-mL aliquot of the rumen fluid-buffer mixture was transferred into a 180-mL serum bottle containing 1.0 g of dietary substrate, which had been dried at 55°C for 48 h and ground through a 1-mm screen using a Wiley mill (Arthur H. Thomas, Philadelphia, PA, United States). The liquid volume was standardized across treatments to maintain consistent buffering capacity, with each bottle receiving the same rumen fluid to buffer ratio (1:2, v/v). Bottles were sealed and incubated at 39°C for 48 h with continuous shaking at 80 rpm. Fermentation was terminated by immersing the bottles in ice water.

### 2.3 Sample collection and chemical analysis

Upon completion of the fermentation, the final pH was determined using a pH meter (Ecoscan pH 5, Singapore). The supernatant from the fermentation fluid was collected and stored at −20°C. The supernatant was then analyzed for volatile fatty acids (VFAs). Ammonia nitrogen (NH₃-N) was quantified using the indophenol method with an acidified procedure, following Weatherburn (19). Lactate concentration was determined using an assay kit, according to the manufacturer’s instructions (Jiancheng Bioengineering Research Institute, Nanjing, China). Microbial crude protein (MCP) was measured using the BCA Protein Assay Kit (Tiandz Inc., Beijing, China). The substrate-fermentation fluid mixture was collected and stored at −80°C for subsequent microbiota analysis. Feed ingredients were analyzed by wet chemistry methods for CP (GB/T 6432-2018), amylase-treated NDF (20), ADF (NY/T 1459-2022), EE (GB/T 6433-2006), ash (GB/T 6438-2007), and calculated NFC (17). The composition and nutrient levels of the experimental diets are presented in Table 1.

Gas production was measured using a pressure transducer (21). Methane (CH_4_) and hydrogen (H_2_) production were quantified using a GC-TCD instrument (Agilent 7890B, Agilent, California, United States). Gasses were separated using packed GC columns (Porapak Q & MolSieve 5A, Agilent, California, United States) under the following conditions: column temperature, 80°C; injection temperature, 200°C; detector temperature, 200°C; carrier gas, N_2_. The VFAs were determined according to Jin et al. (22). Each 1.0 mL sample was mixed with 0.2 mL of deproteinization-acidification solution [25% (w/v) metaphosphoric acid and 0.65% (w/v) crotonic acid] before gas chromatographic analysis (Agilent 7890B, Agilent, California, United States). Separation was performed using a fused silica capillary column (Supelco, Bellefonte, United States) under the following conditions: initial column temperature, 110°C for 3 min; ramping at 40°C/min to 150°C; injection temperature, 200°C; flame ionization detector (FID) temperature, 220°C; carrier gas, N_2_.

### 2.4 DNA extraction and real-time PCR

Genomic DNA was extracted from 1.0 mL of fermentation fluid using bead-beating combined with phenol–chloroform–isoamyl alcohol extraction, as described by Jin et al. (23). The extracted DNA was subsequently divided into two aliquots for downstream sequencing and quantitative real-time PCR (qPCR) analysis.

The abundances of bacteria, archaea, anaerobic fungi, and protozoa were quantified using a 7300 Real-Time PCR System (Applied Biosystems, California, United States). The primers used for these four microbial groups are listed in Supplementary Table S1. The reaction mixture was prepared using SYBR^®^ Premix Ex Taq^™^ (TaKaRa, Dalian, China). DNA copy numbers were determined in triplicate for each sample, and the mean value was calculated. Standard curves were constructed using plasmid DNA containing cloned target genes of the respective microbial groups. Results were expressed as gene copy numbers per milliliter of fermentation fluid.

### 2.5 16S rRNA gene sequencing and data analysis

Microbial DNA was extracted from samples using the E. Z. N. A.^®^ Soil DNA Kit (Omega Bio-Tek, Norcross, GA, United States) following manufacturer’s instructions. Bacterial 16S rRNA genes were amplified using the primer pair 341F (5′-CCTAYGG-GRBGCASCAG-3′) and 806R (5′-GGACTACNNGGGTATCTAAT-3′). Archaeal 16S rRNA genes were amplified using the primer pair Arch519F (5′-CAGCCGCCGCGGTAA-3′) and Arch915R (5′-GTGCTCCCCCGCCAATTCCT-3′). The amplicons were sequenced using paired-end (PE250) sequencing on an Illumina MiSeq platform by BIOZERON Biotechnology Co., Ltd. (Shanghai, China). The raw data were stored in the Sequence Read Archive (SRA) database of the National Biotechnology Information Center (NCBI), https://www.ncbi.nlm.nih.gov/, accession number: PRJNA1014950, bacteria; PRJNA1014951, archaea.

Demultiplexed paired-end reads were imported into QIIME2 (v2020.11), and DADA2 was employed for quality filtering, chimera removal, merging of overlapping paired-end reads, and generation of amplicon sequence variants (ASVs) (24). Trimming and filtering were applied to paired reads with a maximum of two expected errors per read (maxEE = 1). To assess the adequacy of sequencing depth for capturing microbial diversity, rarefaction curves were generated for both bacterial and archaeal communities (Supplementary Figure S1). Taxonomic classification was performed using the RDP Classifier (stand-alone version, RDP Classifier v2.14; https://sourceforge.net/projects/rdp-classifier/), with the SILVA database (v138) for bacterial taxonomy and the RIM database for methanogens. Alpha diversity analysis was conducted using Mothur (v1.21.1). Beta diversity was assessed via principal coordinate analysis (PCoA) based on Bray–Curtis distances. The significance of differences among groups was assessed using ANOSIM in the vegan package of R (v3.6.3).

### 2.6 Statistical analysis

Nutritional digestibility, gas production, and fermentation parameters were analyzed using a randomized complete design, with treatment as a fixed effect and run and run-by-treatment interaction as random effects. The run-by-treatment interaction was used as the error term to test the treatment effect. Linear and quadratic effects of treatments were analyzed using orthogonal contrasts. Microbial data were statistically analyzed using R (v3.6.3). For variables that did not meet the assumption of normality, the Kruskal–Wallis test was applied. Differences were considered statistically significant at *p* < 0.05.

## 3 Results

### 3.1 Gas production and nutrients degradation

Gas production and nutrient degradation rates are presented in Table 2. Total gas production, as well as the degradation rates of DM and NDF, decreased linearly (*p* < 0.05) with increasing NDF/NFC ratio. In contrast, methane yield (mL/g dry matter degradation) increased linearly (*p* < 0.05) with increasing NDF/NFC ratio. The degradation rate of ADF was significantly higher in R0.48 (*p* < 0.05), whereas no significant differences were observed among the remaining groups (*p* > 0.05). No significant effects of treatment were found for the other measured parameters (*p* > 0.05).

**Table 2:** Effects of dietary NDF/NFC ratios on *in vitro* production of gas and nutrients degradation.

Items	Groups^1^					SEM	*p*-value		
	R_0.48_	R_0.57_	R_0.70_	R_0.90_	R_1.12_		Treatment	Linear	Quadratic
Gas production									
Total gas (mL)	205.60^a^	203.60^a^	202.80^a^	190.40^b^	179.80^c^	2.27	<0.001	<0.001	<0.001
Methane (mL)	30.55	31.60	31.14	30.42	29.24	0.29	0.090	0.600	0.018
Methane (mL/g DMD)^2^	35.11^c^	37.18^bc^	37.52^b^	40.02^a^	40.05^a^	0.49	<0.001	<0.001	<0.001
Hydrogen (mL)	0.39	0.44	0.49	0.31	0.30	0.03	0.183	0.129	0.120
Hydrogen (mL/g DMD)	0.45	0.52	0.59	0.40	0.41	0.03	0.391	0.407	0.309
Nutrient degradation (%)									
Dry matter	86.71^a^	85.30^ab^	82.51^b^	75.56^c^	73.22^c^	1.07	<0.001	<0.001	<0.001
Neutral detergent fiber	60.00^a^	50.33^b^	50.11^b^	43.01^c^	37.93^c^	1.76	<0.001	0.005	0.002
Acid detergent fiber	51.05^a^	38.37^b^	40.67^b^	36.69^b^	35.71^b^	1.52	0.002	0.338	0.018

1 R_0.48_ (NDF/NFC = 0.48), R_0.57_ (NDF/NFC = 0.57), R_0.70_ (NDF/NFC = 0.70), R_0.90_ (NDF/NFC = 0.90) and R1.12 (NDF/NFC = 1.12).

2 DMD, dry matter degradation.

### 3.2 *In vitro* fermentation characteristics

Fermentation characteristics are presented in Table 3. The pH of R0.48 was lower than that of R0.90 and R1.12 and increased linearly with increasing NDF/NFC ratio (*p* < 0.05). Acetate and the acetate-to-propionate ratio (A: P) in R0.48 and R0.57 were lower than those in R0.90 and R1.12 and increased linearly with increasing NDF/NFC ratio (*p* < 0.05). Propionate was significantly higher in R0.48 and R0.57 than in R0.90 and R1.12, and it decreased linearly with increasing NDF/NFC ratio (*p* < 0.05). Ammonia nitrogen was higher in R0.57 and R0.70 than in the other groups (*p* < 0.05), whereas no significant difference was observed between R0.57 and R0.70 (*p* > 0.05). Microbial crude protein (MCP) was higher in R0.48 than in R0.90 and R1.12 (*p* < 0.05). Lactate concentration was lower in R0.48 than in the other groups (*p* < 0.05) and increased linearly with the NDF/NFC ratio (*p* < 0.05).

**Table 3:** Fermentation parameters from 48-h *in vitro* fermentation.

Items			Groups^1^			SEM	*P*-value		
	R_0.48_	R_0.57_	R_0.70_	R_0.90_	R_1.12_		Treatment	Linear	Quadratic
pH	6.25^b^	6.35^ab^	6.33^ab^	6.42^a^	6.43^a^	0.02	0.007	<0.001	<0.001
Acetate (mol/100 mol)	53.40^c^	53.59^c^	54.35^bc^	55.24^ab^	56.23^a^	0.27	0.001	<0.001	<0.001
Propionate (mol/100 mol)	29.40^a^	28.73^ab^	28.23^b^	27.08^c^	26.57^c^	0.26	<0.001	<0.001	<0.001
Butyrate (mol/100 mol)	11.97	12.34	12.14	12.31	12.07	0.17	0.820	0.458	0.647
Isobutyrate (mol/100 mol)	1.14	1.15	1.12	1.19	1.02	0.02	0.586	0.002	0.008
Valerate (mol/100 mol)	1.85	1.86	1.84	2.01	2.01	0.06	0.683	0.222	0.484
Isovalerate (mol/100 mol)	2.24^ab^	2.33^a^	2.32^a^	2.17^bc^	2.10^c^	0.02	0.004	0.013	0.001
TVFA (mmol/L)	111.43^a^	110.50^a^	103.63^bc^	105.27^b^	101.70^c^	0.91	<0.001	0.014	0.042
A: P	1.82^c^	1.87^bc^	1.93^b^	2.04^a^	2.12^a^	0.03	<0.001	<0.001	<0.001
Ammonia-nitrogen (mol/L)	37.19^b^	39.80^a^	39.63^a^	36.61^b^	37.25^b^	0.41	0.006	0.266	0.040
MCP (mg/dL)	15.57^a^	15.19^ab^	14.31^bc^	13.68^c^	14.56^bc^	0.18	0.003	0.185	0.150
Lactate (mmol/L)	0.19^c^	0.35^b^	0.31^b^	0.44^b^	0.64^a^	0.17	<0.001	<0.001	<0.001

1 R_0.48_ (NDF/NFC = 0.48), R_0.57_ (NDF/NFC = 0.57), R_0.70_ (NDF/NFC = 0.70), R_0.90_ (NDF/NFC = 0.90) and R1.12 (NDF/NFC = 1.12).

### 3.3 The quantification of bacteria, archaea, anaerobic fungi and protozoa

The results of the real-time PCR analysis are shown in Table 4. No significant differences were observed in the abundance of bacteria, archaea, and protozoa among the five treatment groups (*p* > 0.05). However, both archaeal and protozoal abundances exhibited a significant linear increase with increasing NDF/NFC ratio (*p* < 0.05). The abundance of anaerobic fungi was significantly lower in the R0.48 group (*p* < 0.05), but higher in the R0.90 group compared to R0.48 and R0.57 (*p* < 0.05). Additionally, the abundance of anaerobic fungi increased linearly with the NDF/NFC ratio (*p* < 0.001).

**Table 4:** Microbe numbers from 48 h *in vitro* fermentation.

Items	Groups^1^						*P*-value		
	R_0.48_	R_0.57_	R_0.70_	R_0.90_	R_1.12_	SEM	Treatment	Linear	Quadratic
Bacteria (log_10/_L)	7.80	7.99	7.78	7.71	7.69	0.06	0.554	0.242	0.433
Archaea (log_10_/L)	5.57	5.64	5.62	5.72	5.68	0.02	0.158	0.032	0.093
Anaerobic fungus (log_10_/L)	3.14^c^	3.61^b^	3.77^ab^	3.93^a^	3.80^ab^	0.06	<0.001	<0.001	<0.001
Protozoa (log_10_/L)	3.92	4.10	4.22	4.30	4.25	0.04	0.156	0.002	0.003

1 R_0.48_ (NDF/NFC = 0.48), R_0.57_ (NDF/NFC = 0.57), R_0.70_ (NDF/NFC = 0.70), R_0.90_ (NDF/NFC = 0.90) and R1.12 (NDF/NFC = 1.12).

### 3.4 Rumen microbial community structures

#### 3.4.1 Effects on bacterial community

**Figure 1:**
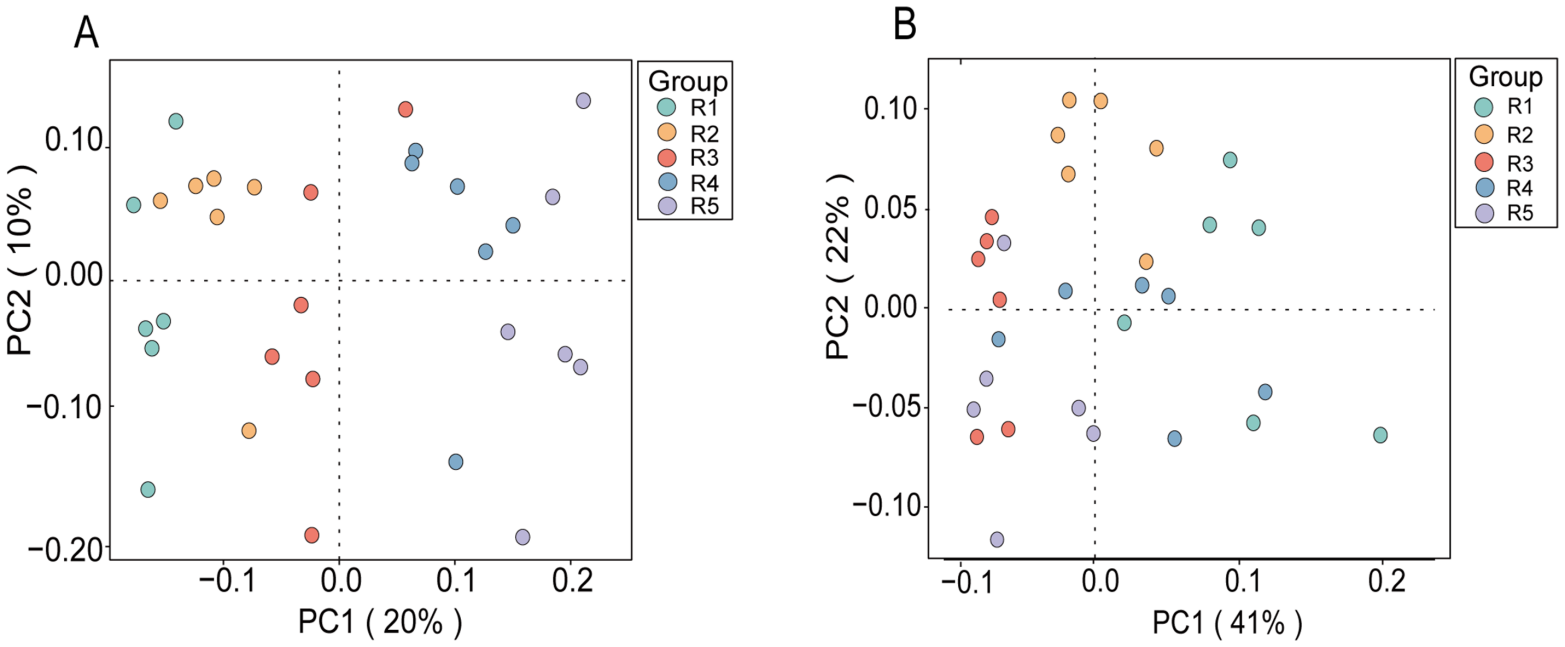
**(A)** PCoA analysis of bacterial populations based on Bray curtis distance, Anosim (*R* = 0.298, *p* = 0.001); **(B)** PCoA analysis of archaeal populations based on Bray curtis distance, Anosim (*R* = 0.470, *p* = 0.001). The colors of the dots in both plots represent different experimental groups (Group); each dot represents an individual sample. R1 = R0.48 (NDF/NFC = 0.48), R2 = R0.57 (NDF/NFC = 0.57), R3 = R0.70 (NDF/NFC = 0.70), R4 = R0.90 (NDF/NFC = 0.90), and R5 = R1.12 (NDF/NFC = 1.12).

A total of 1,129,463 bacterial sequences were remained with an average of 37,648 clean reads per sample after quality filtering (Table 5). The average length was 414 bp. A total of 10,074 ASVs were identified. Alpha diversity results revealed that Chao1, ACE, Shannon and Simpson indices of R0.48 and R0.57 were lower than R1.12 (*p* < 0.05), and the Shannon and Simpson indices were decreased in R0.48 than R0.90 (*p* < 0.05). These four indexes of R0.57 were not significantly different than R0.70 and R0.90 (*p* > 0.05). There was a clear separation of clusters on the PCoA plot among the 5 groups (Anosim, *R* = 0.298, *p* = 0.001, Figure 1A). PC1 and PC2 accounted for 20 and 10% of the total variance, respectively.

**Table 5:** Alpha diversity of bacterial and archaea populations.

Items			Groups^1^			SEM	*P*-value
	R_0.48_	R_0.57_	R_0.70_	R_0.90_	R_1.12_		
Bacteria							
Reads	37240.2	36586.7	35473.8	39392.7	39550.5	486.8	0.052
Chao1	876.5^b^	898.1^b^	887.9^b^	1010.3^ab^	1048.4^a^	15.5	<0.001
ACE	877.05^b^	898.62^b^	888.11^b^	1011.17^ab^	1049.36^a^	15.54	<0.001
Shannon	5.61^c^	5.75^bc^	5.87^abc^	6.14^ab^	6.28^a^	0.05	<0.001
Simpson	0.9862^c^	0.9891^bc^	0.9915^abc^	0.9949^ab^	0.9963^a^	<0.01	<0.001
Archaea							
Reads	37807.8	37748.7	37138.2	38997.3	38267.5	464.5	0.764
Chao1	61.3^a^	52.1^b^	53.0^b^	61.8^a^	61.5^a^	1.2	0.007
ACE	61.44^a^	52.29^b^	53.05^b^	61.87^a^	61.6^a^	1.2500	0.009
Shannon	2.99^a^	2.76^b^	2.75^b^	2.94^ab^	2.87^ab^	0.0200	0.006
Simpson	0.9251^a^	0.9128^ab^	0.9072^b^	0.9227^a^	0.9126^ab^	<0.01	0.004

1 R_0.48_ (NDF/NFC = 0.48), R_0.57_ (NDF/NFC = 0.57), R_0.70_ (NDF/NFC = 0.70), R_0.90_ (NDF/NFC = 0.90) and R1.12 (NDF/NFC = 1.12).

**Figure 2:**
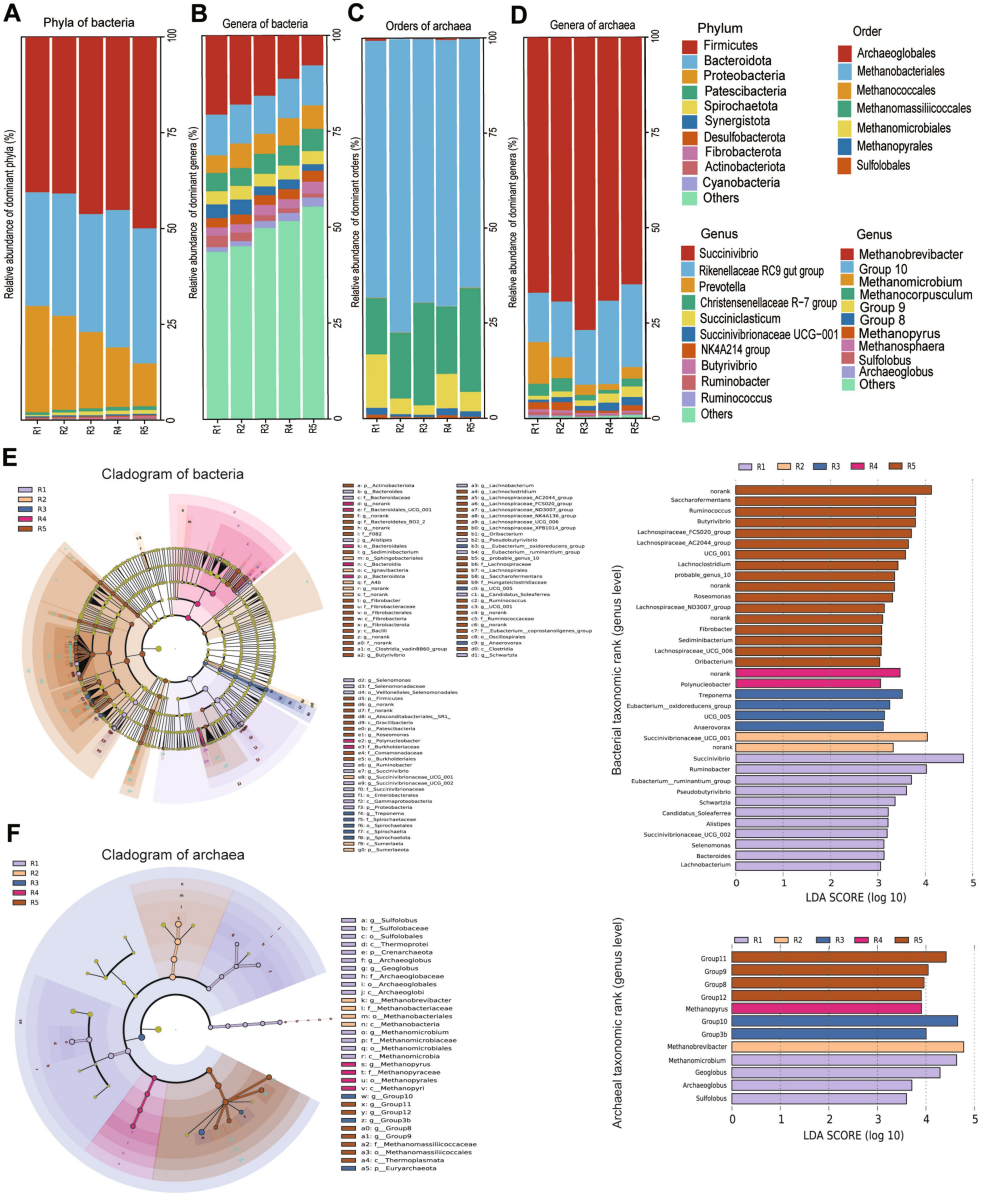
**(A,B)** The relative abundance at the level of phyla and genera in bacteria; **(C,D)** the relative abundance at the level of orders and genera in archaea; Linear discriminant analysis (LDA) effect size (LEfSe) (logarithmic LDA score of_3.0) of the ruminal microbiota. Taxonomic rank labels are provided before bacterial names; p_, c_, o_, f_, and g_indicate phylum, class, order, family, and genus, respectively. The greater the LDA score of the biomarker taxon mean value, the greater the influence of species abundance on the difference in the microbial community in the different treatments. **(E)** LEfSe analysis of the bacterial community showing the difference among groups (*n* = 6 samples per group), LDA scores at the genus level of bacteria; **(F)** LEfSe analysis of the bacterial community showing the difference among groups (*n* = 6 samples per group), LDA scores at the genus level of archaea. R1 = R0.48 (NDF/NFC = 0.48), R2 = R0.57 (NDF/NFC = 0.57), R3 = R0.70 (NDF/NFC = 0.70), R4 = R0.90 (NDF/NFC = 0.90), and R5 = R1.12 (NDF/NFC = 1.12).

At the phylum level, a total of 26 phyla were identified across all samples. The three predominant phyla (the average relative abundances of phyla >1% in at least one group) were Firmicutes (43.12%), Bacteroidota (31.64%), Proteobacteria (19.20%) (Figure 2A). The relative abundance of Firmicutes was significantly reduced in R0.48 and R0.57 than R1.12 (*p* < 0.05), but the difference was not significant compared with other groups (*p* > 0.05). The relative abundance of Bacteroidota in R0.48 was lower than R0.90 (*p* < 0.05). The relative abundance of Proteobacteria in R0.48 and R0.57 was higher than R1.12 group (*p* < 0.05) (Supplementary Table S2).

A total of 437 bacterial genera were identified from all samples. The 10 predominant genera (the average relative abundances of genera >1.5% in at least one group) were *Succinivibrio* (14.26%), *Rikenellaceae RC9 gut group* (9.97%), *Prevotella* (5.68%), *Christensenellaceae R-7 group* (4.98%), *Succiniclasticum* (3.33%), *Succinivibrionaceae UCG-001* (2.72%), *NK4A214 group* (2.52%), *Butyrivibrio* (2.42%), *Ruminobacter* (1.72%), *Ruminococcus* (1.70%) (Figure 2B). LEfSe and LDA analysis showed that *Ruminococcus*, *Pseudobutyrivibrio*, *[Eubacterium] ruminantium group*, *Saccharofermentans* of Firmicutes phylum and *Succinivibrio*, *Succinivibrionaceae* UCG-001, *Ruminobacter* of Proteobacteria phylum caused the differences in bacterial communities among the 5 groups (Figure 2E, LDA score > 4). The relative abundance of *Succinivibrio*, *Succinivibrionaceae* UCG-001, *Ruminobacter* in R0.48 and R0.57 was significantly higher than R1.12 (*p* < 0.05). The relative abundance of *Ruminococcus* and *Saccharofermentans* in R1.12 was higher than R0.48 and R0.57 (*p* < 0.05) (Supplementary Table S3).

#### 3.4.2 Effects on archaeal community

A total of 1,139,757 archaeal sequences were remained with an average of 37,991 clean reads per sample after quality filtering (Table 5). The average length was 382 bp. A total of 5,995 ASVs were identified. Alpha diversity results revealed that Chao1 and ACE indices of R0.48, R0.90 and R1.12 were higher than R0.57 and R0.70 (*p* < 0.05). The Shannon index was increased in R0.48 than R0.57 and R0.70 (*p* < 0.05), but there was no significant difference than the other groups (*p* > 0.05). Simpson index of R0.48 was higher than R0.70 (*p* < 0.05), but not significantly different from the other groups (*p* > 0.05, Table 5). There was a clear separation of clusters on the PCoA plot among the 5 groups (Anosim, *R* = 0.470, *p* = 0.001, Figure 1B). PC1 and PC2 accounted for 41 and 22% of the total variance, respectively.

At the order level, a total of 7 orders were identified across all samples. The three predominant orders (the average relative abundance of orders > 1% in at least one group) were Methanobacteriales (34.67%), Methanomassiliicoccales (10.71%) and Methanomicrobiales (2.63%, Figure 2C). Methanobacteriales, Methanomassiliicoccales and Methanomicrobiales caused the differences in archaeal communities among the 5 groups. The relative abundance of Methanobacteriales in R0.57 and R0.70 was higher compared with R0.48 (*p* < 0.05). The relative abundance of Methanomicrobiales in R0.48 was higher than R0.70 (*p* < 0.05), but Methanomassiliicoccales was higher in R0.70 than R0.48 and R0.90 (*p* < 0.05) (Supplementary Table S4).

A total of 23 archaeal genera were identified. The three predominant genera (the average relative abundances of genera >1% in at least one group) were *Methanobrevibacter* (34.37%), *Group10* (8.74%) and *Methanomicrobium* (1.76%, Figure 2D). The results of LEfSe and LDA analysis also showed that these three archaea mainly affected the diversity of archaea community (Figure 2F, LDA score > 4). The relative abundance of *Methanobrevibacter* in R0.57 and R0.70 was higher than R0.48 (*p* < 0.05). The relative abundance of *Group10* in R0.70 was higher than R0.48 and R0.90 (*p* < 0.05). The relative abundance of *Methanomicrobium* in R0.48 was higher than R0.70 and R1.12 (*p* < 0.05) (Supplementary Table S5).

#### 3.4.3 Correlation between taxa and VFAs

**Figure 3:**
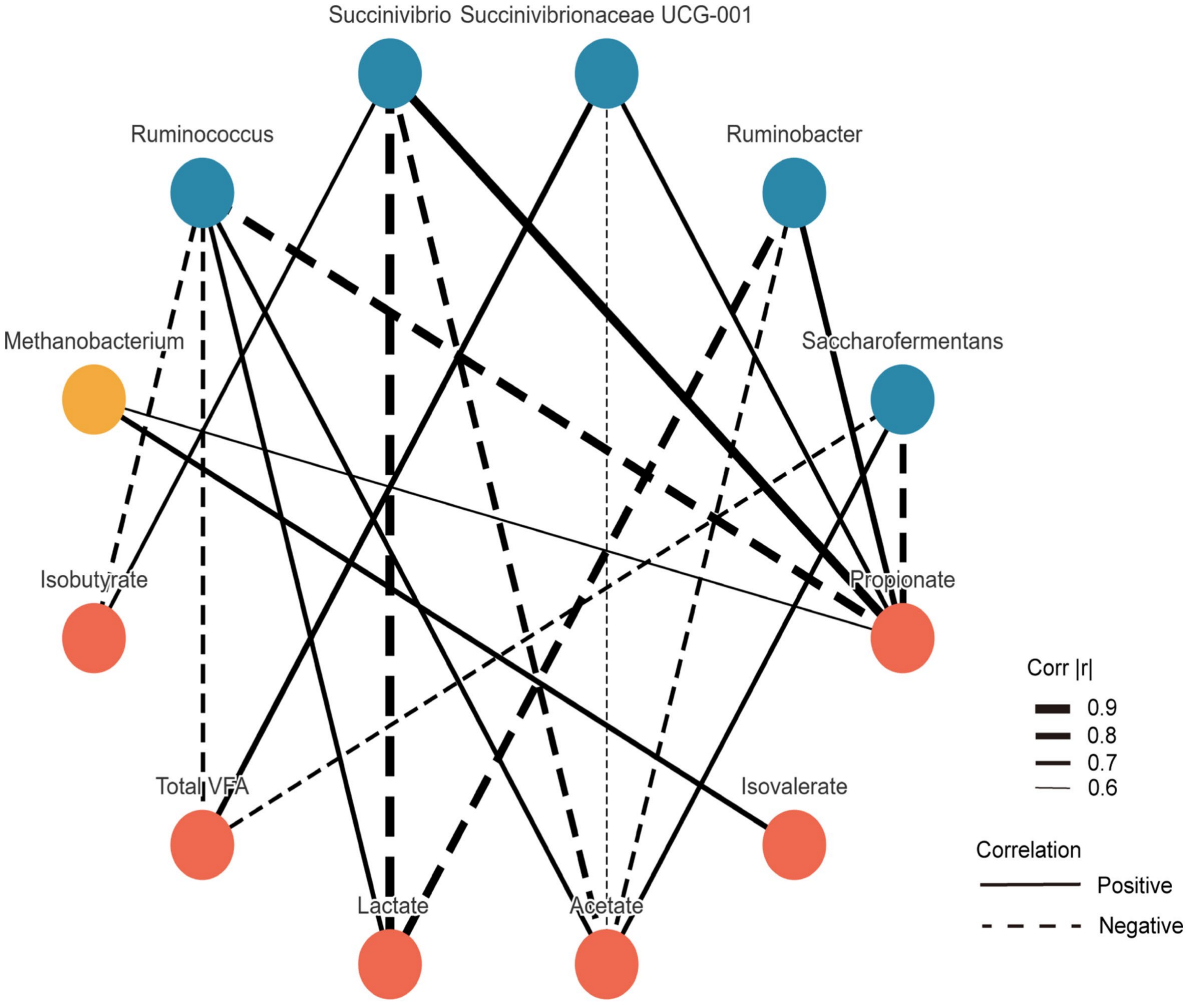
Spearman’s correlation analysis among differential taxa at the genus level. Only features showing strong significant correlations (|*r*| > 0.5 and *p* < 0.05) were visualized.

The correlations between microbial taxa and VFAs are illustrated in Figure 3. The relative abundance of *Succinivibrio* was negatively correlated with acetate and lactate (*p* < 0.05), but positively correlated with propionate and isobutyrate (*p* < 0.05). *Succinivibrionaceae UCG-001* abundance showed a negative correlation with acetate (*p* < 0.05), and positive correlations with propionate and total VFAs (TVFA) (*p* < 0.05). Similarly, *Ruminobacter* abundance was negatively correlated with acetate and lactate, but positively associated with propionate (*p* < 0.05). In contrast, *Ruminococcus* exhibited negative correlations with propionate, isobutyrate, and TVFA (*p* < 0.05), while being positively correlated with acetate and lactate (*p* < 0.05). Additionally, the relative abundance of *Saccharofermentans* was negatively correlated with propionate and TVFA (*p* < 0.05), and positively correlated with acetate (*p* < 0.05).

## 4 Discussion

In this study, five dietary formulations were designed with varying neutral detergent fiber to non-fiber carbohydrate (NDF/NFC) ratios, while maintaining an identical crude protein content across all treatments. Although no statistically significant differences were observed in total methane production among the five diets, a slight upward trend was noted in treatments with NDF/NFC ratios of 0.57 and 0.70. Interestingly, methane production per unit of dry matter degraded increased with rising NDF/NFC ratios, which aligns with findings from a previous study on *in vitro* rumen fermentation using substrates with varying metabolizable energy levels (25). Unfortunately, the study did not provide data on NFC, although the NDF levels ranged from 42.2 to 60.4%. In contrast, the current study, despite lower NDF levels (22.9–40.7%), yet a similar trend was observed, indicating a strong relationship between methane production and the extent of DM degradation. In contrast, the present study, despite having lower NDF levels (22.9–40.7%), demonstrated a similar trend to that reported by a previous study (25), highlighting a strong association between methane production and the extent of dry matter degradation.

Conversely, in another *in vitro* rumen fermentation study with two forage-to-concentrate ratio diets (30:70; 70:30), the degradation rate of DM and methane production were lower in the high forage (high NDF) diet (26). These findings are inconsistent with the results of the current study. Methane production per unit of DM degraded may be influenced by both the composition of the substrates and their degradation rates. For instance, substrates containing slowly fermentable starch tend to produce higher methane yields compared to those with rapidly fermentable starch (27). Furthermore, a diet with a low NDF/NFC ratio produces less hydrogen per unit of fermented carbohydrates, leading to reduced methane production (26, 28). However, in the present study, the pH values across all groups were maintained at relatively high levels, which did not significantly affect the fermentation process. In the group with the lower NDF/NFC ratio, the higher degradation rates of both DM and NDF led to a greater total amount of fermented carbohydrates, likely ensuring a sufficient hydrogen supply for methanogenesis and thereby explaining the absence of significant differences in total methane production among the dietary treatments. Notably, although total methane production remained relatively stable, methane yield per unit of degraded DM (CH₄/DMD) increased significantly with higher NDF/NFC ratios. This observation can be attributed to both altered fermentation patterns and reduced microbial utilization efficiency. A marked increase in the acetate-to-propionate ratio (from 1.82 to 2.12) reflected a fermentative shift toward pathways that generate more hydrogen (29), while concurrent declines in TVFA concentration and MCP production suggested reduced microbial growth and lower efficiency of energy capture. Together, these factors likely led to an accumulation of available hydrogen, which was subsequently utilized by methanogens, thus explaining the elevated CH₄/DMD ratio. These results indicate that the increased methane yield per unit of degraded substrate was driven by a combination of greater hydrogen availability and inefficient microbial fermentation under high-fiber dietary conditions.

Previous studies have reported that high concentrate feeding (characterized by low NDF content) tend to reduce ruminal pH, which in turn reduces NDF digestibility (30). However, this finding contrasts with the results of the current study. As the NDF/NFC ratios increased, the degradation rates of DM, NDF, and ADF decreased. This discrepancy may be attributed to differences in rumen pH. The pH did not decline to excessively low levels by the end of fermentation, ranging from 6.25 to 6.43. This stability in pH likely had a negligible or minimal impact on fermentation, ensuring that the fermentation process was not significantly disrupted. The relatively stable pH results may be due to the buffering agents present in the culture medium used for *in vitro* rumen fermentation.

As the NDF/NFC ratio increased, the proportion of acetate increased, while that of propionate declined, consistent with findings from previous studies (31–33). The concentration of TVFA was higher in the lower NDF/NFC groups, which aligned with the higher degradation rate of DM. Lactate concentration was higher in the higher NDF/NFC groups. Lactate can be converted to propionate in the rumen (34), but there may have been factors that hindered this conversion in the current study. The lowest NDF/NFC diet resulted in the highest MCP production. Under consistent dietary protein levels, a diet with higher energy content promotes MCP synthesis (35, 36), with the observed increase in VFA concentrations in this study indicating more efficient feed breakdown (37), thereby enhancing VFA yields and providing greater energy for MCP synthesis. The abundance of bacteria archaea and protozoa was not affected by the NDF/NFC ratios. Anaerobic fungi, which are key fiber-degrading microbes in the rumen, were promoted by diets with higher NDF content (38–40).

The NDF/NFC ratios altered the bacterial community composition. The bacterial genera *Succinivibrio*, *Succinivibrionaceae* UCG-001, and *Ruminobacter* were enriched in the lower NDF/NFC groups, with their relative abundance positively correlated with propionate concentration. *Succinivibrio* and *Succinivibrionaceae* can produce succinate and a small amount of lactate, both of which serve as precursors of propionate (41). Moreover, the enrichment of *Succinivibrionaceae* UCG-001 was observed in the rumen of cattle fed a high-grain diet (42). In contrast, *Ruminococcus* and *Saccharofermentans* were enriched in the higher NDF/NFC groups and displayed a negative correlation with the propionate concentration. These two bacterial taxa are involved in fiber degradation (43, 44). *Ruminococcus*, belonging to the Firmicutes phylum, degrades dietary fiber and regulates acetate and butyrate concentrations while producing acetate and hydrogen (45, 46). *Ruminococcus* can produce acetate and hydrogen. Additionally, *Ruminococcus* modulates microbial activity, potentially contributing significantly to rumen fermentation (47). *Saccharofermentans* can produce hydrogen peroxide, fumarate, lactate, and acetate when fermenting starch or other carbohydrates (48). The observed changes in these bacterial populations are closely linked to the alterations in propionate concentration. As a hydrogen sink, propionate production reduces the availability of hydrogen, which reduces methane formation.

The NDF/NFC ratios significantly altered the archaeal community composition, with distinct implications for methane biosynthesis pathways in the rumen. Moderate NDF/NFC ratios (R0.57 and R0.70) increased the relative abundance of *Methanobrevibacter*, consistent with a prior *in vitro* study where its copy number was higher in a balanced 50% alfalfa and 50% concentrate diet compared to extreme diets (49). This suggests that moderate NDF/NFC ratios enhance hydrogen availability, supporting *Methanobrevibacter* growth and methane production. Conversely, the low NDF/NFC ratio (R0.48) enriched *Methanomicrobiales*, which also contributes to hydrogenotrophic methanogenesis but may thrive on rapidly fermentable substrates, as reported by Friedman et al. (50). A dairy cow study similarly reported higher *Methanomicrobiales* abundance at an NDF/NFC ratio of 0.71 compared to 1.02 (51), indicating that low NDF/NFC diets favor this group. Notably, the R0.70 group showed increased *Methanomassiliicoccales* (Group 10), which utilize methyl compounds (e.g., methanol, methylamines) for methylotrophic methanogenesis (29). This increase may be linked to pectin-derived methyl compounds in moderate NDF diets, though this requires further validation. Variability in *Methanobrevibacter* responses across studies, with no changes in *Karakul* sheep (52) and increased abundance in goats at lower NDF/NFC ratios (53), likely reflects dietary composition and host-specific factors. Functionally, lower NDF/NFC ratios in our study enhanced propionate production via Succinivibrio, reducing H₂ availability for hydrogenotrophic methanogenesis and lowering methane yields (2). In contrast, moderate NDF/NFC ratios supported fibrolytic bacteria (*Ruminococcus*), increasing H₂ and methane via *Methanobrevibacter* (29). Thus, NDF/NFC ratios shape archaeal communities and methane biosynthesis pathways, offering a strategic approach to mitigate emissions.

## 5 Conclusion

The dietary NDF/NFC ratios influenced the *in vitro* rumen fermentation and microbiota. The NDF/NFC ratios did not affect the total methane production. As the NDF/NFC ratios increased, the methane yield per unit of dry matter degraded, the concentration of TVFA and acetate increased, while the concentration of propionate decreased. Higher NDF/NFC ratios promoted the growth of anaerobic fungi and altered the bacterial and archaeal populations. The findings of this study provide a theoretical basis for reducing methane emissions through adjusting the dietary NDF/NFC ratios.

## Data Availability

The raw data were stored in the Sequence Read Archive (SRA) database of the National Biotechnology Information Center (NCBI), https://www.ncbi.nlm.nih.gov/, accession number: PRJNA1014950, bacteria; PRJNA1014951, archaea.
