# Supplementary material for: Effects of dietary NDF/NFC ratios on in vitro rumen fermentation, methane emission, and microbial community composition
Source: Front Vet Sci. 2025 Jun 24;12:1588357. doi: 10.3389/fvets.2025.1588357 (PMC12235747; doi:10.3389/fvets.2025.1588357)
Supplement: Supplementary file 1 [file Table_1.docx]

**Table S1** The real-time PCR primers used in this study

| Microbes | Primers | Sequences | Annealing temperature (°C) | Reference |
| --- | --- | --- | --- | --- |
| Bacteria (189 bp) | F  R | CCTACGGGAGGCAGCAG  ATTACCGCGGCTGCTGG | 60 | Metzler-Zebeli et al. (1) |
| Archaea (420 bp) | F  R | GTGCTCCCCCGCCAATTCCT  GCGGTGTGTGCAAGGAGC | 59 | Jeyanathan et al. (2) |
| Protozoa (223 bp) | F  R | GCTTTCGWTGGTAGTGTATT  CTTGCCCTCYAATCGTWCT | 55 | Sylvester et al. (3) |
| Fungi (120 bp) | F  R | GAGGAAGTAAAAGTCGTAACAAGGTTTC  CAAATTCACAAAGGGTAGGATGATT | 60 | Denman et al. (4) |

Reference:

1. Metzler-Zebeli BU, Schmitz-Esser S, Klevenhusen F, Podstatzky-Lichtenstein L, Wagner M, Zebeli Q. Grain-Rich Diets Differently Alter Ruminal and Colonic Abundance of Microbial Populations and Lipopolysaccharide in Goats. *Anaerobe.* (2013) 20: 65-73. doi: 10.1016/j.anaerobe.2013.02.005.
2. Jeyanathan J, Kirs M, Ronimus RS., Hoskin SO, Janssen PH. Methanogen Community Structure in the Rumens of Farmed Sheep, Cattle and Red Deer Fed Different Diets. *FEMS Microbiol. Ecol.* (2011) 76(2): 311-26. doi: 10.1111/j.1574-6941.2011.01056.x.
3. Sylvester JT, Karnati SKR, Yu ZT, Morrison M, Firkins JL. Development of an Assay to Quantify Rumen Ciliate Protozoal Biomass in Cows Using Real-Time PCR. *J. Nutr.* (2004) 134(12): 3378-84. doi: 10.1093/jn/134.12.3378.
4. Denman SE, McSweeney CS. Development of a Real-Time PCR Assay for Monitoring Anaerobic Fungal and Cellulolytic Bacterial Populations Within the Rumen. *FEMS. Microbiol. Ecol.* (2006) 58(3): 572-82. Doi: 10.1111/j.1574-6941.2006.00190.x.
