# Supplementary material for: Effects of dietary NDF/NFC ratios on in vitro rumen fermentation, methane emission, and microbial community composition
Source: Front Vet Sci. 2025 Jun 24;12:1588357. doi: 10.3389/fvets.2025.1588357 (PMC12235747; doi:10.3389/fvets.2025.1588357)
Supplement: Supplementary file 2 [file Table_2.docx]

**Table S2** Effect of different NDF/NFC Ratios of dietary on phylum-level diversity (the relative abundance >1%) in the bacterial community.

| Items | R_0.48_ | R_0.57_ | R_0.70_ | R_0.90_ | R_1.12_ | SEM | *P*-value |
| --- | --- | --- | --- | --- | --- | --- | --- |
| Firmicutes | 39.97^b^ | 39.77^b^ | 44.89^ab^ | 43.08^ab^ | 47.9^a^ | 0.73 | 0.001 |
| Bacteroidota | 29.27^b^ | 31.07^ab^ | 29.90^ab^ | 34.21^a^ | 33.76^ab^ | 0.59 | 0.019 |
| Proteobacteria | 27.31^a^ | 23.86^ab^ | 19.32^abc^ | 14.81^bc^ | 10.72^c^ | 1.15 | 0.000 |
| Others | 3.45^b^ | 5.30^ab^ | 5.89^ab^ | 7.90^a^ | 7.62^a^ | 0.41 | 0.004 |

R_0.48_ (NDF/NFC =0.48), R_0.57_ (NDF/NFC =0.57), R_0.70_ (NDF/NFC =0.70), R_0.90_ (NDF/NFC =0.90) and R1.12 (NDF/NFC =1.12).
