# Supplementary material for: Effects of dietary NDF/NFC ratios on in vitro rumen fermentation, methane emission, and microbial community composition
Source: Front Vet Sci. 2025 Jun 24;12:1588357. doi: 10.3389/fvets.2025.1588357 (PMC12235747; doi:10.3389/fvets.2025.1588357)
Supplement: Supplementary file 3 [file Table_3.docx]

**Table S3** Effect of different NDF/NFC Ratios of dietary on genus-level diversity (the relative abundance >1% in at least one group) in the bacterial community.

| Items | R_0.48_ | R_0.57_ | R_0.70_ | R_0.90_ | R_1.12_ | SEM | *P*-value |
| --- | --- | --- | --- | --- | --- | --- | --- |
| Proteobacteria |  |  |  |  |  |  |  |
| *Succinivibrio* | 20.29^a^ | 17.49^ab^ | 15.25^abc^ | 10.79^bc^ | 7.52^c^ | 0.91 | <0.001 |
| *Succinivibrionaceae UCG-001* | 3.51^a^ | 3.80^a^ | 2.23^ab^ | 2.40^ab^ | 1.63^b^ | 0.22 | 0.004 |
| *Ruminobacter* | 2.93^a^ | 2.16^ab^ | 1.44^abc^ | 1.14^bc^ | 0.92^c^ | 0.16 | <0.001 |
| Bacteroidota |  |  |  |  |  |  |  |
| *Rikenellaceae RC9 gut group* | 10.49 | 9.90 | 9.66 | 9.79 | 10.01 | 0.19 | 0.617 |
| *Prevotella* | 4.50 | 6.20 | 5.04 | 6.80 | 5.85 | 0.38 | 0.213 |
| *Christensenellaceae R-7 group* | 4.71 | 4.54 | 5.08 | 4.99 | 5.57 | 0.12 | 0.148 |
| *Muribaculaceae_norank* | 2.99 | 2.58 | 3.34 | 2.64 | 2.76 | 0.17 | 0.689 |
| *p-251-o5_norank* | 0.92 | 1.09 | 1.18 | 1.73 | 1.56 | 0.12 | 0.128 |
| *Bacteroidales RF16 group_norank* | 1.32 | 1.21 | 1.09 | 1.16 | 1.31 | 0.04 | 0.525 |
| *Prevotellaceae UCG-003* | 0.93 | 1.20 | 0.76 | 1.18 | 1.04 | 0.08 | 0.249 |
| Firmicutes |  |  |  |  |  |  |  |
| *Succiniclasticum* | 3.36 | 3.46 | 3.16 | 3.45 | 3.24 | 0.27 | 0.886 |
| *NK4A214 group* | 2.40 | 2.46 | 2.47 | 2.50 | 2.75 | 0.08 | 0.590 |
| *Butyrivibrio* | 2.14 | 2.12 | 2.59 | 2.27 | 3.00 | 0.11 | 0.061 |
| *Ruminococcus* | 1.15^c^ | 1.28^bc^ | 1.77^abc^ | 2.01^ab^ | 2.28^a^ | 0.10 | <0.001 |
| *Pseudobutyrivibrio* | 1.96^a^ | 1.78^a^ | 1.73^a^ | 1.40^ab^ | 1.18^b^ | 0.07 | 0.001 |
| *[Eubacterium] ruminantium group* | 2.10^a^ | 1.87^a^ | 1.42^ab^ | 1.10^b^ | 1.19^b^ | 0.08 | <0.001 |
| *Clostridia UCG-014_norank* | 1.34 | 1.33 | 1.36 | 1.56 | 1.62 | 0.05 | 0.270 |
| *Saccharofermentans* | 0.90^c^ | 1.06^bc^ | 1.49^abc^ | 1.54^ab^ | 2.05^a^ | 0.08 | <0.001 |
| *Lachnospiraceae_Unclassified* | 1.63^a^ | 1.34^ab^ | 1.56^a^ | 1.09^ab^ | 1.05^b^ | 0.06 | 0.002 |
| *UCG-010_norank* | 1.13 | 1.25 | 1.34 | 1.40 | 1.49 | 0.05 | 0.168 |
| *Lachnospiraceae_uncultured* | 1.13 | 1.14 | 1.34 | 1.08 | 1.34 | 0.04 | 0.122 |
| *UCG-005* | 1.09ab | 0.96b | 1.24a | 1.06ab | 1.14ab | 0.03 | 0.022 |

R_0.48_ (NDF/NFC =0.48), R_0.57_ (NDF/NFC =0.57), R_0.70_ (NDF/NFC =0.70), R_0.90_ (NDF/NFC =0.90) and R1.12 (NDF/NFC =1.12).
