# Supplementary material for: Effects of dietary NDF/NFC ratios on in vitro rumen fermentation, methane emission, and microbial community composition
Source: Front Vet Sci. 2025 Jun 24;12:1588357. doi: 10.3389/fvets.2025.1588357 (PMC12235747; doi:10.3389/fvets.2025.1588357)
Supplement: Supplementary file 4 [file Table_4.docx]

**Table S4** Effect of different NDF/NFC Ratios of dietary on order-level diversity (the relative abundance >1%) in the archaeal community.

| Items | R_0.48_ | R_0.57_ | R_0.70_ | R_0.90_ | R_1.12_ | SEM | *P*-value |
| --- | --- | --- | --- | --- | --- | --- | --- |
| Methanobacteriales | 22.93^b^ | 45.26^a^ | 45.73^a^ | 28.16^ab^ | 31.25^ab^ | 2.29 | 0.002 |
| Methanomassiliicoccales | 5.15^b^ | 10.27^ab^ | 17.52^a^ | 7.60^b^ | 12.99^ab^ | 0.98 | 0.001 |
| Methanomicrobiales | 4.31^a^ | 2.13^ab^ | 1.49^b^ | 3.00^ab^ | 2.20^ab^ | 0.29 | 0.014 |

R_0.48_ (NDF/NFC =0.48), R_0.57_ (NDF/NFC =0.57), R_0.70_ (NDF/NFC =0.70), R_0.90_ (NDF/NFC =0.90) and R1.12 (NDF/NFC =1.12).
