# Supplementary material for: Effects of dietary NDF/NFC ratios on in vitro rumen fermentation, methane emission, and microbial community composition
Source: Front Vet Sci. 2025 Jun 24;12:1588357. doi: 10.3389/fvets.2025.1588357 (PMC12235747; doi:10.3389/fvets.2025.1588357)
Supplement: Supplementary file 5 [file Table_5.docx]

**Table S5** Effect of different NDF/NFC Ratios of dietary on genus-level diversity (the relative abundance >1% in at least one group) in the archaeal community.

| Items | R_0.48_ | R_0.57_ | R_0.70_ | R_0.90_ | R_1.12_ | SEM | *P*-value |
| --- | --- | --- | --- | --- | --- | --- | --- |
| *Methanobrevibacter* | 22.69^b^ | 44.90^a^ | 45.41^a^ | 27.89^ab^ | 30.99^ab^ | 2.28 | 0.002 |
| *Group10* | 4.50^b^ | 8.45^ab^ | 14.17^a^ | 6.20^b^ | 10.36^ab^ | 0.77 | 0.001 |
| *Methanomicrobium* | 3.36^a^ | 1.43^ab^ | 0.93^b^ | 1.83^ab^ | 1.27^b^ | 0.17 | ＜0.001 |

R_0.48_ (NDF/NFC =0.48), R_0.57_ (NDF/NFC =0.57), R_0.70_ (NDF/NFC =0.70), R_0.90_ (NDF/NFC =0.90) and R1.12 (NDF/NFC =1.12).
