# Supplementary material for: Effects of dietary NDF/NFC ratios on in vitro rumen fermentation, methane emission, and microbial community composition
Source: Front Vet Sci. 2025 Jun 24;12:1588357. doi: 10.3389/fvets.2025.1588357 (PMC12235747; doi:10.3389/fvets.2025.1588357)
Supplement: Supplementary file 6 [file Table_6.docx]

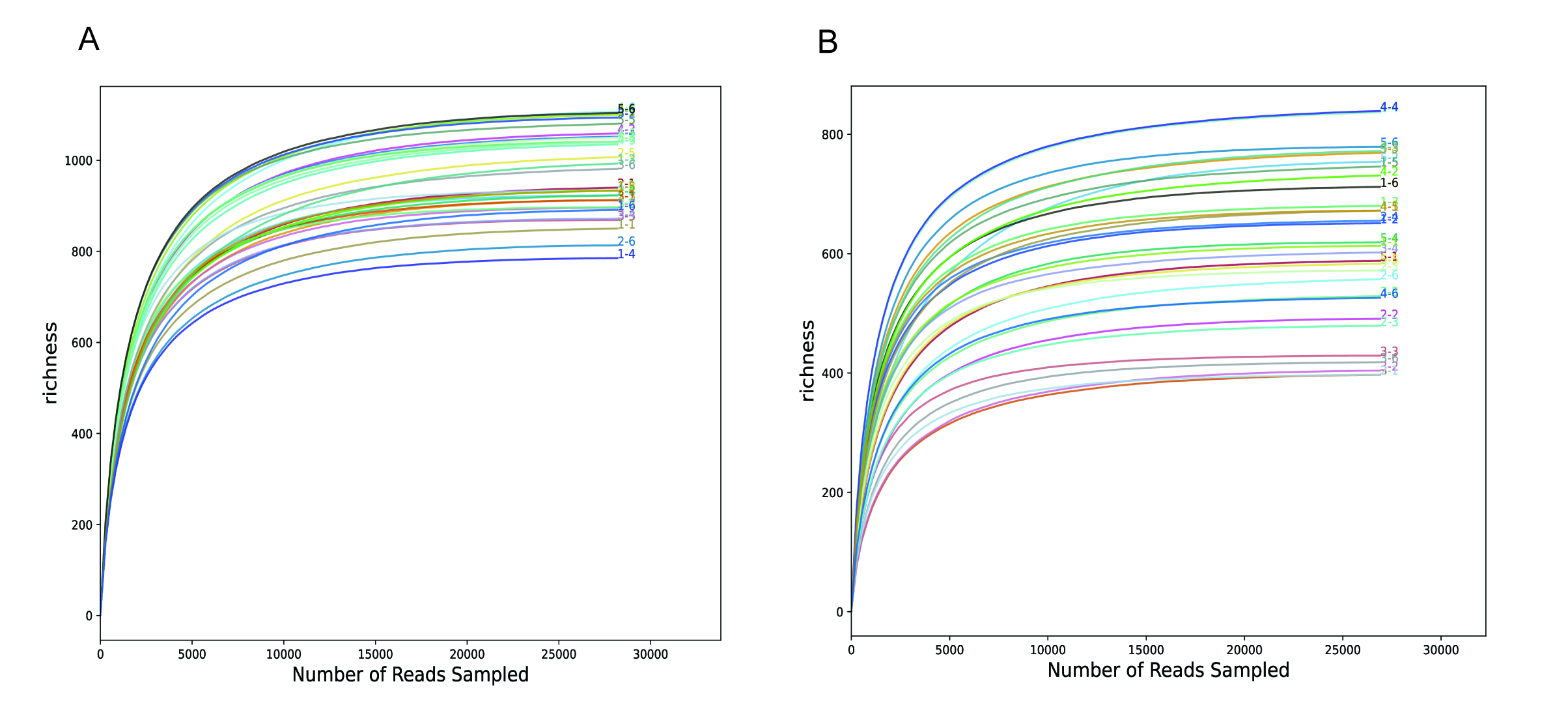


**Figure S1**. Rarefaction curves demonstrating microbial diversity sampling saturation across sequencing effort for **(A)** bacterial and **(B)** archaeal communities. The x - axis represents the number of sequencing reads randomly subsampled from each specimen, while the y - axis denotes the corresponding count of ASVs (Amplicon Sequence Variants) recovered at each sequencing depth. This rarefaction curve visualizes the relationship between sequencing effort and resolution of microbial diversity, with distinct trajectories color-coded by sample to assess whether sufficient sequencing depth was achieved for community characterization.
